# Pulmonary inflammation-induced alterations in key regulators of mitophagy and mitochondrial biogenesis in murine skeletal muscle

**DOI:** 10.1186/s12890-020-1047-8

**Published:** 2020-01-21

**Authors:** Pieter A. Leermakers, Alexander H. V. Remels, Ramon C. J. Langen, Annemie M. W. J. Schols, Harry R. Gosker

**Affiliations:** 1 Department of Respiratory Medicine, NUTRIM School of Nutrition and Translational Research in Metabolism, Maastricht University Medical Centre+, P.O. Box 5800, 6202 AZ Maastricht, the Netherlands; 2 Department of Pharmacology and Toxicology, NUTRIM School of Nutrition and Translational Research in Metabolism, Maastricht University Medical Centre+, Maastricht, the Netherlands

**Keywords:** Inflammation, Mitophagy, Mitochondrial biogenesis, Skeletal muscle

## Abstract

**Background:**

Both mitophagy, a selective mechanism for clearance of mitochondria, and mitochondrial biogenesis are key processes determining mitochondrial content and oxidative capacity of the musculature. Abnormalities in these processes could therefore contribute to deterioration of peripheral muscle oxidative capacity as observed in e.g. chronic obstructive pulmonary disease. Although it has been suggested that inflammatory mediators can modulate both mitophagy and mitochondrial biogenesis, it is unknown whether acute pulmonary inflammation affects these processes in oxidative and glycolytic skeletal muscle in vivo. Therefore, we hypothesised that molecular signalling patterns of mitochondrial breakdown and biogenesis temporally shift towards increased breakdown and decreased biogenesis in the skeletal muscle of mice exposed to one single bolus of IT-LPS, as a model for acute lung injury and pulmonary inflammation.

**Methods:**

We investigated multiple important constituents and molecular regulators of mitochondrial breakdown, biogenesis, dynamics, and mitochondrial content in skeletal muscle over time in a murine (FVB/N background) model of acute pulmonary- and systemic inflammation induced by a single bolus of intra-tracheally (IT)-instilled lipopolysaccharide (LPS). Moreover, we compared the expression of these constituents between gastrocnemius and soleus muscle.

**Results:**

Both in soleus and gastrocnemius muscle, IT-LPS instillation resulted in molecular patterns indicative of activation of mitophagy. This coincided with modulation of mRNA transcript abundance of genes involved in mitochondrial fusion and fission as well as an initial decrease and subsequent recovery of transcript levels of key proteins involved in the molecular regulation of mitochondrial biogenesis. Moreover, no solid differences in markers for mitochondrial content were found.

**Conclusions:**

These data suggest that one bolus of IT-LPS results in a temporal modulation of mitochondrial clearance and biogenesis in both oxidative and glycolytic skeletal muscle, which is insufficient to result in a reduction of mitochondrial content.

## Background

Chronic obstructive pulmonary disease (COPD) is characterised by skeletal muscle mitochondrial impairments, which contribute to a decreased quality of life and survival [1]. During the course of the disease, COPD patients suffer from episodic increases in respiratory symptoms, i.e. acute exacerbations, which are often caused by pulmonary infections and are associated with acute pulmonary inflammation [2, 3]. Since COPD exacerbations are associated with accelerated muscle deterioration, which includes increased protein breakdown and the transcriptional impairments in the oxidative phenotype [1, 4], it is likely that mitochondrial deterioration is accelerated during exacerbations as well.

Processes involved in remodelling and homeostasis of the mitochondrial network include biogenesis of new organelles, mitochondrial clearance by mitophagy, and fusion and fission events [5].

Mitochondrial breakdown via selective autophagy (i.e. mitophagy), a process defined by selective autophagy of mitochondria [6], has received much attention in recent years as an essential mechanism of mitochondrial quality control and remodelling, and can roughly be divided in receptor-mediated mitophagy and ubiquitin-mediated mitophagy [7]. Receptor-mediated mitophagy is regulated through activation of mitophagy-receptors like BCL2/adenovirus E1B 19 kDa protein interacting protein 3 (BNIP3), BNIP3 like (BNIP3L), or FUN14 domain containing 1 (FUNDC1), while ubiquitin-mediated mitophagy is activated by a loss of mitochondrial membrane potential and is initiated by the recruitment/activation of proteins like PTEN induced putative kinase 1 (PINK1) and Parkin (PARK2). Both these pathways eventually result in the recruitment of general autophagy proteins to facilitate autophagosomal membrane formation around the mitochondrion and predestines the organelle for degradation [8–13]. Mitochondrial biogenesis, on the other hand, is essentially controlled by the peroxisome proliferative activated receptor (PPAR), gamma, coactivator 1 (PPARGC1) signalling network, which is comprised of many different transcription factors regulating the coordinated transcription of nuclear- and mitochondrial-DNA encoded metabolic genes [14].

It is clear from studies in experimental sepsis models that severe acute systemic inflammation negatively impacts mitochondrial health and content, and results in mitophagy, autophagy, and decreased mitochondrial biogenesis in skeletal muscles [15–22]. Moreover, patients with sepsis have a lower mitochondrial content than age-matched controls [23, 24]. It is therefore feasible that the inflammatory component of the acute exacerbation of COPD affects muscle mitochondria in a similar manner. Although previous studies show that bacterial-induced pulmonary infection and aspiration pneumonia indeed result in the upregulation of markers for autophagy in skeletal muscles [25, 26], an extensive characterisation of the mitochondrial homeostasis pathways has currently not yet been performed in a model for pulmonary-inflammation induced systemic inflammation.

Therefore, we hypothesised that molecular signalling patterns of mitochondrial breakdown and biogenesis temporally shift towards increased breakdown and decreased biogenesis in the skeletal muscle of mice exposed to acute pulmonary inflammation. To study this hypothesis, we used intra-tracheal (IT)-instillation of lipopolysaccharide (LPS) as an established in vivo model for acute (bacterially originated) pulmonary inflammation. The inflammatory profile of this model has been extensively characterised, and not only comprises of prominent inflammation in the pulmonary compartment, but also in increased circulatory levels of inflammatory mediators as well as activation of inflammatory signalling in the peripheral musculature [27–30]. As secondary objectives, we explored if the hypothesised shift in mitochondrial homeostasis signalling was associated with an actual loss of mitochondrial content, and if the signalling patterns were similar in predominantly glycolytic and oxidative muscles.

## Methods

### Ethical approval

Animal procedures were performed in accordance with the European Directive 2010/63/EU guidelines and conform the journal regulations [31]. Both studies were performed under a protocol approved by the Institutional Animal Care Committee of Maastricht University (DEC-2007-040) in accordance to the National Institutes of Health guide for the care and use of Laboratory animals.

### Experimental animals

The presented murine data concerns data from the genetic control group, expressing 6xhis/GFP-tagged ubiquitin on a FVB/N background (kindly provided by Douglas Gray [32]), of a larger research project concerning multiple unrelated hypotheses [33], in order to reduce number of laboratory animals used. The mice were owned and bred by the animal care facility at Maastricht University. Twelve week old male mice were housed in the animal care facility at Maastricht University with multiple animals (± 4) per cage (with embedding material and cage enrichment) in a temperature-controlled environment with 12 h/12 h light-dark cycle and fed ad libitum. Mice health and body weight was checked daily according to standardized procedures of the animal care facility. Mice were anesthetized using intraperitoneal injection (i.p.) xylazine (3 mg/kg) and ketamine (75 mg/kg), and randomly subjected to one intra-tracheal instillation of 20 μg lipopolysaccharide (IT-LPS) or saline (IT-NaCl) in a random order, after which they were directly allowed to awaken. Mice were sacrificed after 7, 24, 48, 72, 96, or 120 h by i.p. sodium pentobarbital (115 mg/kg) followed by exsanguination. The number of animals used in the time-course study was 58 with group sizes as follows, IT-NaCl 7 h *n* = 3, IT-LPS 7 h *n* = 5, IT-NaCl 24-120 h *n* = 4, and IT-LPS 24-120 h *n* = 6, and the number of animals used in the muscle comparison was 11 with group sizes as follows, IT-NaCl 48 h n = 5, and IT-LPS 48 h *n* = 6. Gastrocnemius and soleus muscles were excised, snap-frozen in liquid nitrogen, and crushed into powder. A small proportion of data included in this manuscript (i.e. proportions of LC3B and SQSTM1 protein and mRNA expression data in the time-course study) has been previously published in a research project concerning unrelated hypotheses [33].

### RNA extraction and qPCR

RNA was extracted from 15 mg muscle powder using TRI Reagent®-based separation methods (Sigma-Aldrich, Zwijndrecht, the Netherlands). Two hundred nanograms of RNA was used for cDNA synthesis using the tetro cDNA synthesis kit (Bioline, Alphen aan de Rijn, the Netherlands) according to manufacturer’s protocol. 4.4 μl of 1/50 diluted cDNA was used for quantitative PCR amplification using target and species specific primers (Table 1) and 2X Sensimix™ SYBR® & Fluorescein mix (Bioline, Alphen aan de Rijn, the Netherlands) on a LightCycler480 384-wells PCR machine (Roche, Almere, the Netherlands). Specificity of PCR amplification was tested with melt curve analysis and expression levels of genes of interest were corrected using a normalization factor calculated based on the expression of 3 different housekeepers (*Rplp0, Rpl13A and B2m*) using the GeNorm software (Primerdesign, Southampton, USA).

**Table 1:** Primers used for qPCR

	Sense primer	Antisense primer	Ensembl identifier
*Rplp0*	GGACCCGAGAAGACCTCCTT	GCACATCACTCAGAATTTCAATGG	ENSMUSG00000067274
*Rpl13A*	CACTCTGGAGGAGAAACGGAAGG	GCAGGCATGAGGCAAACAGTC	ENSMUSG00000074129
*B2m*	CTTTCTGGTGCTTGTCTCACTGA	GTATGTTCGGCTTCCCATTCTC	ENSMUSG00000060802
*Cxcl1*	TCGTCTTTCATATTGTATGGTCAACACG	TGCCCTACCAACTAGACACAAAATGTC	ENSMUSG00000029380
*Tnf*	GGGCCACCACGCTCTTC	TACAGGCTTGTCACTCGAATTTTG	ENSMUSG00000024401
*Cxcl2*	CCCTGGTTCAGAAAATCATCCAAA	TTTGGTTCTTCCGTTGAGGGAC	ENSMUSG00000058427
*Nfkbia*	GCTACCCGAGAGCGAGGAT	GCCTCCAAACACACAGTCATCA	ENSMUSG00000021025
*Bnip3*	AGGTTTTCCTTCCATCTCTGTTACTG	TGTGTGAACAGAAGTCAGATCCAAA	ENSMUSG00000078566
*Bnip3l*	AGTCGGGACAGAGCAGCTCAAG	TCAAACATGATCTGCCCATCTTCTT	ENSMUSG00000022051
*Fundc1*	CGAGTATTTGGCCACAGTTCC	CCACTGTGACTGGCAACCTG	ENSMUSG00000025040
*Pink1*	GTCCTGAAGGGAGCAGACG	TTAAGATGGCTTCGCTGGAG	ENSMUSG00000028756
*Park2*	CTGGCTGTCCCAACTCCCT	CCTCGGCCCCATACTGC	ENSMUSG00000023826
*Optn*	GAGCTGCAGGTGGAGAGCAT	CCACCTTTTCTGCCTGTTGC	ENSMUSG00000026672
*Ndufb3*	ACAGACAGTGGAAAATTGAAGGG	GCCCATGTATCTCCAAGCCT	ENSMUSG00000026032
*Sdhb*	AATTTGCCATTTACCGATGGGA	AGCATCCAACACCATAGGTCC	ENSMUSG00000009863
*Cyc1*	GCATTCGGAGGGGTTTCCAG	CCGCATGAACATCTCCCCA	ENSMUSG00000022551
*mt-Cox2*	CCATCCCAGGCCGACTAA	ATTTCAGAGCATTGGCCATAGAA	ENSMUSG00000064354
*Ppargc1a*	CAACAATGAGCCTGCGAACA	CTTCATCCACGGGGAGACTG	ENSMUSG00000029167
*Ppargc1b*	ACCCTGAGAAAGCGCAATGA	CCCAGATGAGGGAAGGGACT	ENSMUSG00000033871
*Ppara*	ACTACGGAGTTCACGCATGTG	TTGTCGTACACCAGCTTCAGC	ENSMUSG00000022383
*Ppard*	AGGCCCGGAGCATCCTCA	TGGATGACAAAGGGTGCGTTG	ENSMUSG00000002250
*Tfam*	CCGGCAGAGACGGTTAAAAA	TCATCCTTTGCCTCCTGGAA	ENSMUSG00000003923
*Nrf1*	AGCCACATTGGCTGATGCTT	GGTCATTTCACCGCCCTGTA	ENSMUSG00000058440
*Gabpa*	TGCTGCACTGGAAGGCTACA	TTACCCAAACCACCCAATGC	ENSMUSG00000008976
*Esrra*	GGCGGACGGCAGAAGTACAAA	GCGACACCAGAGCGTTCAC	ENSMUSG00000024955
*Dnm1l*	TGCCCACTGAGCAATCTCAA	TGCTAACACATTTAGGCAGTGTGTACT	ENSMUSG00000022789
*Fis1*	GGGCAACTACCGGCTCAAG	GCCATGCCTACCAGTCCATC	ENSMUSG00000019054
*Mtfp1*	CCACCACACTTGGACTGCTG	GGCTTCTCCACTGACGGGTA	ENSMUSG00000004748
*Opa1*	GGGAAAACCAGTGTGCTGGA	AACAAGGCCACATGGTGAGG	ENSMUSG00000038084
*Mfn1*	GCTGGCTGTCTTGTGCATGT	TCCAGCTCTGTGGTGACATCTG	ENSMUSG00000027668
*Mfn2*	CAGGGGTATCAGCGAAGTGC	ACCAATCCCAGATGGCAGAA	ENSMUSG00000029020
*Lc3b*	GAGCAGCACCCCACCAAGAT	CGTGGTCAGGCACCAGGAA	ENSMUSG00000031812
*Gabarapl1*	CCCTCCCACCAGTGCTACCAT	TCATCACTGTAGGCCACATACAGAAAA	ENSMUSG00000030161
*Sqstm1*	GCAGCTGCTCTTCGGAAGTC	CCCACCGACTCCAAGGCTAT	ENSMUSG00000015837

### Protein extraction and Western blotting

Fifteen milligrams muscle powder was homogenized in 350–400 μl IP lysis buffer (50 mM Tris, 150 mM NaCl, 10% glycerol, 0.5% Nonidet P40, 1 mM EDTA, 1 mM Na_3_VO_4_, 5 mM NaF, 10 mM β-glycerophosphate, 1 mM Na_4_O_7_P_2_, 1 mM DTT, 10 μg/μl leupeptin, 1% apropeptin, 1 mM PMSF, pH 7.4) with the Polytron PT 1600 E (Kinematica, Luzern, Switzerland). Lysates were incubated while rotating for 30 min, and subsequently centrifuged at 20.000 x g for 30 min at 4 °C. Protein concentrations were determined using the Pierce™ BCA Protein Assay Kit (Thermo Scientific, Landsmeer, #23225) according to the manufacturer’s protocol. Lysate (1 μg/μl) was aliquoted in sample buffer (0.25 M Tris-HCl, 8% (w/v) SDS, 40% (v/v) glycerol, 0.4 M DTT, 0.04% (w/v) Bromphenol Blue, pH 6.8) and boiled for 5 min at 95 °C.

Ten micrograms of protein per sample was run through a Criterion 26-wells 12% precast gel (Bio-Rad Laboratories B.V., Veenendaal, the Netherlands) in 1x MES buffer (Bio-Rad Laboratories B.V., Veenendaal, the Netherlands) at 100 V, and was subsequently blotted on a Nitrocellulose membrane (Bio-Rad Laboratories B.V., Veenendaal, the Netherlands) by electroblotting. At least two protein ladders were loaded on each gel (Precision Plus Protein™ All Blue Standards, Bio-Rad Laboratories B.V., Veenendaal, the Netherlands, Bio-Rad Laboratories B.V., #161–0373).

Membranes were incubated in Ponceau S ((Sigma-Aldrich, Zwijndrecht, the Netherlands) for 5 min and washed with milliQ before they were imaged using the LAS-3000 (Fujifilm Life Sciences B.V., Tilburg, the Netherlands) or the Amersham™ Imager 600 (GE Healthcare Life Sciences, Eindhoven, the Netherlands). Total protein Ponceau S quantification was used as correction for gel-loading. Subsequently, membranes were washed, blocked with 3% non-fat, dried milk (Campina, Amersfoort, the Netherlands) in TBS-Tween-20 (0.05%) for 1 h, washed, and incubated overnight at 4 °C with different protein-specific primary antibodies against: TFAM (Millipore Cat# DR1071, RRID:AB_10682431), NRF1 (Abcam Cat# ab55744, RRID:AB_2154534), PPARGC1A (Calbiochem Cat# 516557, RRID:AB_565833), BNIP3 (Cell Signalling Technology Cat# 3769S, RRID:AB_2259284), BNIP3L (Cell Signalling Technology Cat# 12396, RRID:AB_2688036), LC3B (Cell Signalling Technology Cat# 2775, RRID:AB_915950), SQSTM1 (Cell Signalling Technology Cat# 5114, RRID:AB_10624872), PARK2 (Cell Signalling Technology Cat# 4211, RRID:AB_2159920), DNM1L (Cell Signalling Technology Cat# 8570, RRID:AB_10950498), FUNDC1 (Santa Cruz Biotechnology Cat# sc-133,597, RRID:AB_10609242), GABARAPL1 (Proteintech Group Cat# 11010–1-AP, RRID:AB_2294415), oxidative phosphorylation (OXPHOS) complex subunits (MitoScience LLC Cat# MS604, RRID:AB_2629281), AMPK (Cell Signalling Technology Cat# 5832, RRID:AB_10624867), p-AMPK (Thr172) (Cell Signalling Technology Cat# 2535, RRID:AB_331250), ACC (Cell Signalling Technology Cat# 3676, RRID:AB_2219397), and p-ACC (Ser79) (Cell Signalling Technology Cat# 11818, RRID:AB_ 2,687,505) all diluted in 3% non-fat, dried milk or bovine serum albumin in TBS-Tween-20. Membranes were washed and incubated with HRP-labelled, primary antibody-specific, secondary antibody (#BA-9200, #BA-1000, Vector Laboratories, Amsterdam, the Netherlands) (1:10,000 diluted in 3% non-fat, dried milk in TBS-Tween-20) for 1 h at room temperature.

Membranes were washed and incubated with either 0.5x SuperSignal West Pico Chemiluminescent Substrate or 0.25x SuperSignal West Femto Chemiluminescent Substrate (Thermo Scientific, Landsmeer, the Netherlands) for 5 min, depending on the expected signal strength. Photographs were taken with the LAS-3000 or Amersham™ Imager 600 and analysed with ImageQuant TL software (GE Healthcare Life Sciences, Eindhoven, the Netherlands).

### Enzyme activity assays

Fifteen milligrams muscle powder was mixed and homogenized in 240 μl SET buffer (250 mM sucrose, 2 mM EDTA, 10 mM Tris, pH 7.4) using the Mini-BeadBeater (Biospec, Bartlesville, U.S.A.) for 30 s. The solution was snap frozen in liquid nitrogen, defrosted and incubated on ice for 30 min, and subsequently centrifuged at 20,000 x g for 10 min at 4 °C. Twelve microliters 10% BSA was added to 108 μl supernatant (resulting in 1% BSA end concentration), which was stored at − 80 °C. Protein concentration was determined by the Pierce™ BCA Protein Assay Kit in the remaining supernatant.

Enzymatic assays were performed as described previously for both the citrate synthase (CS) assay and β-hydroxyacyl-CoA dehydrogenase (HADH) assay. Absorbance at specific wavelengths was measured using the Multiscan Spectrum machine (Thermo Lab Systems, Landsmeer, the Netherlands) [34]. Slope determination was performed, and corrected for total protein concentration.

### Mitochondrial DNA copy number

Total DNA was extracted from 15 mg muscle powder using the GenElute Mammalian Genomic DNA Miniprep kit (Sigma-Aldrich, Zwijndrecht, the Netherlands) according to manufacturer’s protocol. 4.4 μl 1/10 diluted DNA was used for qPCR as described above, using mitochondrial DNA (mtDNA) or genomic DNA (gDNA) -specific primers (Table 2). mtDNA/gDNA ratio was determined by dividing relative quantity of mtDNA by the relative quantity of gDNA.

**Table 2:** Primers used for assessment of mitochondrial DNA copy number

	Sense primer	Antisense primer	Ensembl identifier
*mt-Nd1*	CAGGATGAGCCTCAAACTCC	GGTCAGGCTGGCAGAAGTAA	ENSMUSG00000064341
*B2m*	GGGAAGTCTTAGGGAGGAGCA	AGCTCTCAAGAACTGTGCCC	ENSMUSG00000060802

### Statistics

Data are depicted as box plots indicating median and interquartile range, with whiskers indicating minimum and maximum, as absolute numbers or as fold change compared to time-matched IT-NaCl. Data from IT-LPS mice was compared with the data form IT-NaCl mice within individual time-points only, using a Mann-Whitney U test. Samples were not subjected to repeated hypotheses. All statistical analyses were performed using IBM SPSS 22 software.

## Results

### Regulation of mitophagy in skeletal muscle in response to IT-LPS

**Fig. 1:**
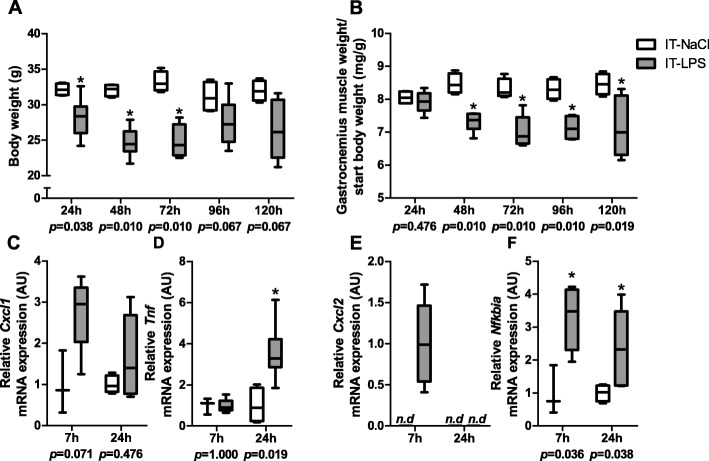
Body weight, m. gastrocnemius weight and *m. gastrocnemius* inflammatory mRNA expression. Body weight (**a**), m. gastrocnemius weight corrected for starting body weight (**b**). mRNA expression levels of *Cxcl1* (**c**), *Tnf* (**d**), *Cxcl2* (**e**) and *Nfkbia* (**f**) in *m. gastrocnemius* are depicted. Data is presented as box plots indicating median and interquartile range, with whiskers indicating min and max. n.d. = not detectable. *P*-value and significant differences are depicted between groups within each time-point *p < 0.05

In line with previous work, IT-LPS instillation in our study resulted in significant reductions in body- as well as muscle weight in the first few days post instillation, indicative of the successful instillation of LPS [27] (Fig. 1a-b). In addition, we observed increased transcript levels of several inflammatory genes in m.gastrocnemius in response to IT-LPS (Fig. 1c-f).

**Fig. 2:**
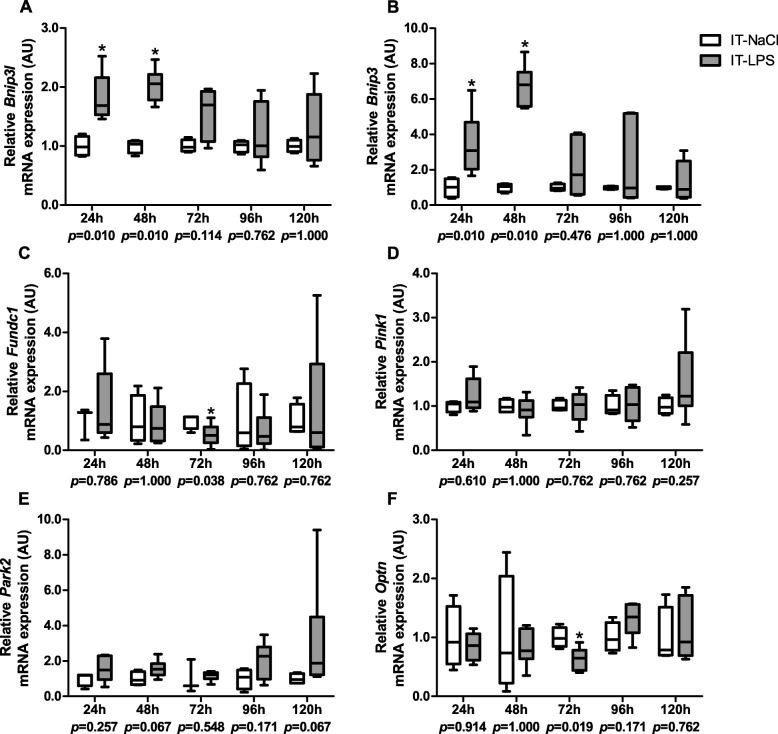
Mitophagy-associated mRNA expression in m. gastrocnemius in response to IT-LPS. mRNA expression levels of *Bnip3l* (**a**), *Bnip3* (**b**), *Fundc1* (**c**), *Pink1* (**d**), *Park2* (**e**) and *Optn* (**f**) in m. gastrocnemius are depicted. Data is presented as box plots indicating median and interquartile range, with whiskers indicating min and max. *p*-value and significance is depicted between groups within each time-point *p < 0.05

To determine the impact of a single bolus of IT-LPS on key processes regulating mitochondrial content, we first assessed mRNA transcript levels and protein abundance of key proteins involved in mitophagy in gastrocnemius muscle at several time-points after IT-LPS instillation. In the first few days post instillation, muscle transcript levels of *Bnip3* and *Bnip3l* were significantly higher in mice subjected to IT-LPS compared with to IT-NaCl-instilled animals (Fig. 2a-b). In addition, muscle *Fundc1* and *Optineurin (Optn)* transcript levels were lower 72 h post instillation, while *Pink1* and *Park2* mRNA levels were largely unaltered at all time-points in the LPS-subjected mice compared with the control group (Fig. 2c-f).

**Fig. 3:**
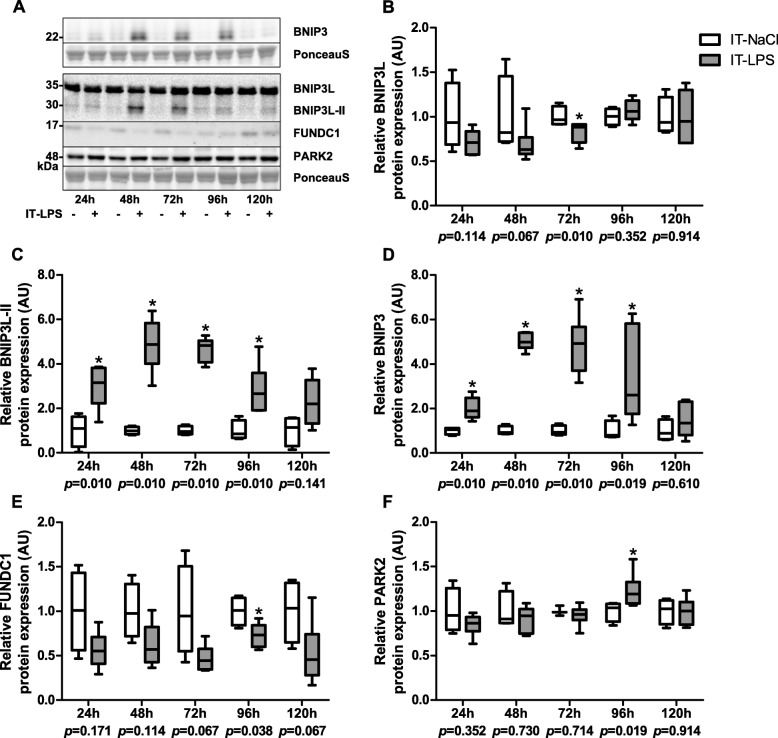
Mitophagy-associated protein expression in m. gastrocnemius in response to IT-LPS. Protein levels of BNIP3L (**b**), BNIP3L-II (**c**), BNIP3 (**d**), FUNDC1 (**e**), and PARK2 (**f**) in m. gastrocnemius are depicted. Representative immunoblots and a representative part of Ponceau S staining are shown, with adjusted contrast equally applied to whole photograph (**a**). Samples were equally divided over multiple gels which were derived and processed in parallel. Data is presented as box plots indicating median and interquartile range, with whiskers indicating min and max. *p*-value and significance is depicted between groups within each time-point *p < 0.05

Western blot analysis revealed two distinct bands for the BNIP3L protein. The band corresponding with the expected molecular weight was termed BNIP3L, while the extra band was termed BNIP3L-II. Specificity of both bands was verified by using a BNIP3L-specific siRNA construct (data not shown). While BNIP3L protein levels in skeletal muscle of mice subjected to IT-LPS were lower compared with controls 72 h post instillation, BNIP3L-II protein levels were found to be significantly higher 24 h–96 h post instillation in the IT-LPS mice, with the largest difference observed 48 h post instillation (Fig. 3ab-c). BNIP3 protein levels showed a similar pattern in response to IT-LPS (Fig. 3d). In addition, FUNDC1 protein levels were significantly lower and PARK2 protein levels were significantly higher at respectively 72 h and 96 h post instillation in the IT-LPS mice (Fig. 3e-f).

**Fig. 4:**
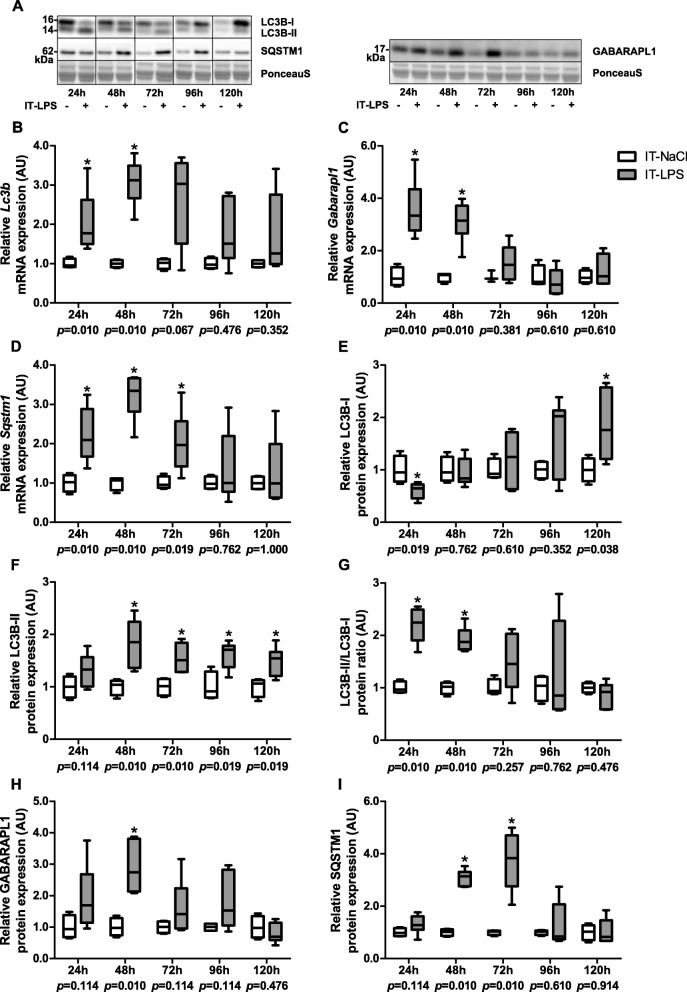
Autophagy-associated protein and mRNA expression in m. gastrocnemius in response to IT-LPS. mRNA expression levels of *Lc3b* (**b**), *Gabarapl1* (**c**) and *sqstm1* (**d**) in m. gastrocnemius are depicted. Protein levels of LC3B (**e-g**), GABARAPL1 (**h**) and SQSTM1 (**i**) in m. gastrocnemius are depicted. Representative immunoblots and a representative part of Ponceau S staining are shown, with cropped photographs indicated by black boxes, with adjusted contrast equally applied to whole photograph (**a**). For LC3B and SQSTM1, samples were grouped by time-point over different gels which were corrected for loading controls. For GABARAPL1, all samples were equally divided over multiple gels which were derived and processed in parallel. Data is presented as box plots indicating median and interquartile range, with whiskers indicating min and max. *p*-value and significance is depicted between groups within each time-point *p < 0.05

Since mitophagy requires several general autophagy-related proteins for generating the autophagosomal membrane and priming the autophagosome to the mitochondria, we studied these proteins as well. In the first days post instillation, transcript levels of the genes coding for the BNIP3 binding partner microtubule- associated protein 1 light chain 3 beta (MAP1LC3B or LC3B) (24 h, 48 h), the BNIP3L binding partner gamma-aminobutyric acid (GABA) A receptor-associated protein-like 1 (GABARAPL1) (24 h, 48 h), and the PINK1/PARK2-related autophagy receptor sequestosome-1 (SQSTM1) (24 h–72 h) were significantly higher in mice subjected to IT-LPS compared with mice subjected to IT-NaCl (Fig. 4a-d). Moreover, the ratio of LC3B-II/LC3B-I protein (24 h, 48 h) as well as protein levels of GABARAPL1 (48 h), and SQSTM1 (48 h, 72 h) were higher during the first days post instillation in the IT-LPS mice compared with the control group (Fig. 4e-i).

### Regulation of mitochondrial biogenesis in skeletal muscle in response to IT-LPS

**Fig. 5:**
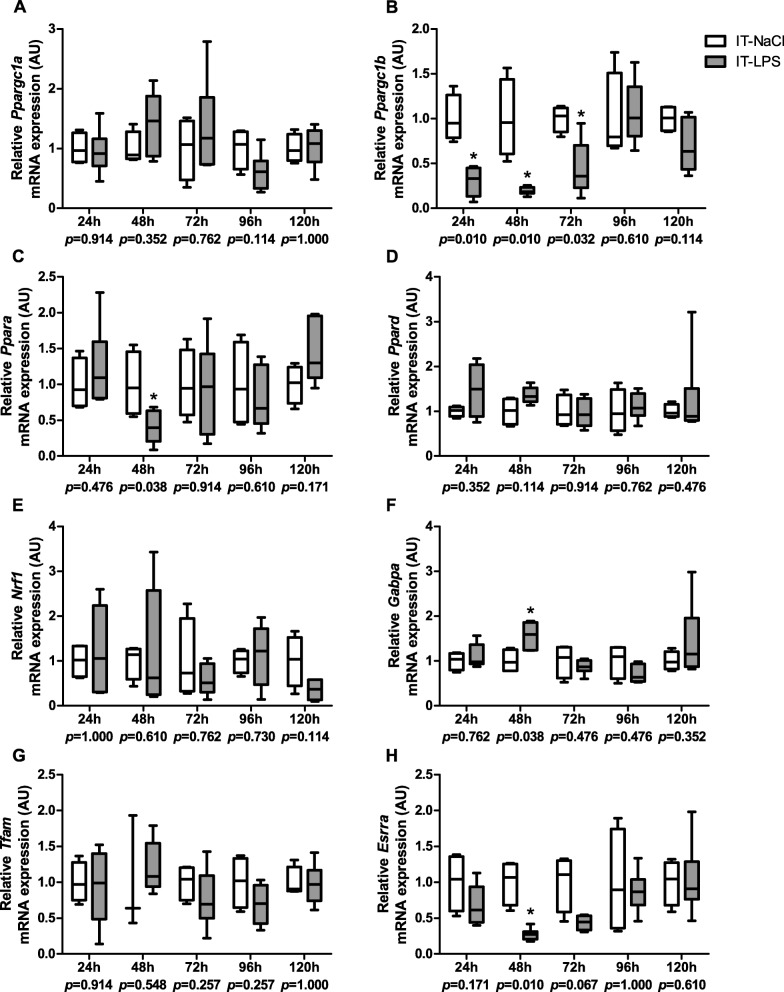
mRNA expression of key regulators of mitochondrial biogenesis in m. gastrocnemius in response to IT-LPS. mRNA expression levels of *Ppargc1a* (**a**), *Ppargc1b* (**b**)*, Ppara* (**c**)*, Ppard* (**d**)*, Nrf1* (**e**)*, Gabpa* (**f**), *Tfam* (**g**)*,* and *Esrra* (**h**) in m. gastrocnemius are depicted. Data is presented as box plots indicating median and interquartile range, with whiskers indicating min and max. *p*-value and significance is depicted between groups within each time-point *p < 0.05

**Fig. 6:**
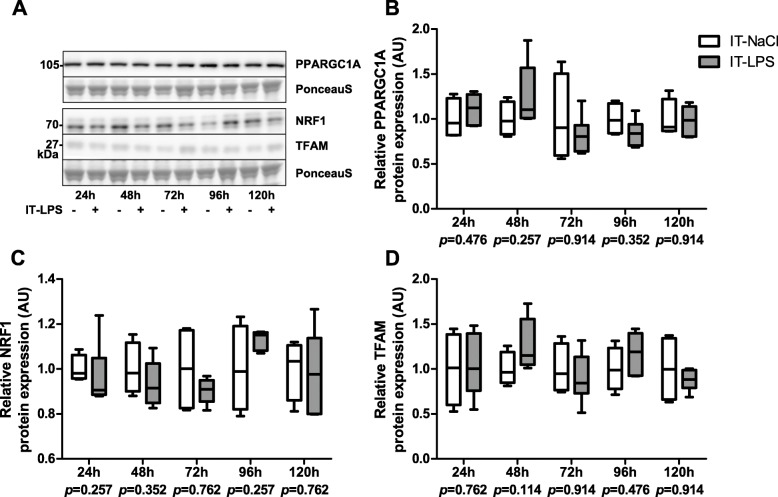
Protein expression of regulators of mitochondrial biogenesis in m. gastrocnemius in response to IT-LPS. Protein expression of PPARGC1A (**b**), NRF1 (**c**) and TFAM (**d**) in m. gastrocnemius are depicted. Representative immunoblots and a representative part of Ponceau S staining are shown, with adjusted contrast equally applied to whole photograph (**a**). Samples were equally divided over multiple gels which were derived and processed in parallel. Data is presented as box plots indicating median and interquartile range, with whiskers indicating min and max. *p*-value and significance is depicted between groups within each time-point *p < 0.05

In order to assess whether IT-LPS instillation impacts the PPARGC1 network in peripheral muscle, we subsequently measured transcript and protein levels of some of its key constituents. *Ppargc1-alpha (Ppargc1a)* transcript levels were largely unchanged while *Ppargc1-beta (Ppargc1b)* transcript levels were dramatically lower in the IT-LPS group in the first few days post instillation (Fig. 5a-b). Moreover, mice subjected to IT-LPS displayed lower transcript levels of *Ppar-alpha (Ppara)* and *estrogen related receptor, alpha (Esrra)*, and higher transcript levels of *GA repeat binding protein, alpha (Gabpa)* 48 h post instillation. No differences in mRNA abundance of *transcription factor A, mitochondrial (Tfam), Ppar-delta (Ppard)* or *nuclear respiratory factor 1 (Nrf1)* were observed at any time-point (Fig. 5c-h). In addition, no differences were found in protein levels of PPARGC1A, NRF1 and TFAM in IT-LPS-treated animals compared with controls at any time-point (Fig. 6a-d).

**Fig. 7:**
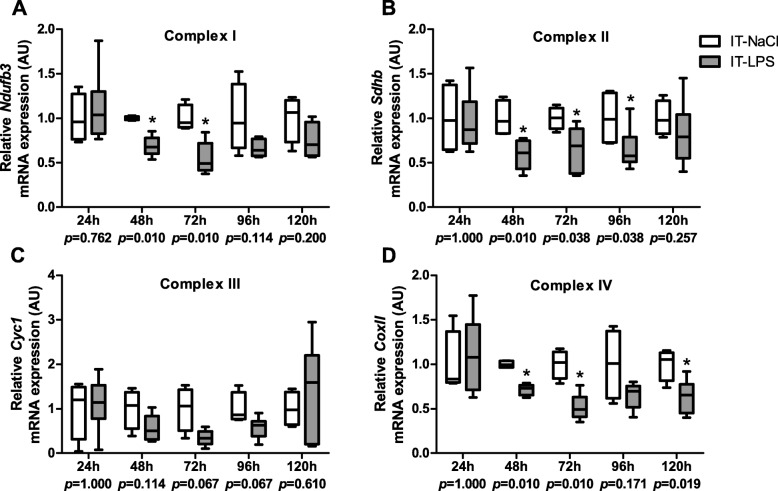
OXPHOS subunit mRNA expression in m. gastrocnemius in response to IT-LPS. mRNA expression levels of the following OXPHOS subunits in m. gastrocnemius are depicted; *Ndufb3* of complex I (**a**), *Sdhb* of complex II (**b**), *Cyc1* of complex III (**c**), and *mt-Co2* of complex IV (**d**). Data is presented as box plots indicating median and interquartile range, with whiskers indicating min and max. *p*-value and significance is depicted between groups within each time-point *p < 0.05

Subsequently, we studied if the abovementioned changes in expression levels of key regulators of mitochondrial biogenesis were associated with changes in transcript levels of different OXPHOS subunits. Indeed, transcript levels of all tested nuclear-encoded OXPHOS subunits (complex I-III) as well as the mitochondrial-encoded OXPHOS subunit Cox II (Complex IV) were, or tended to be, lower in mice subjected to IT-LPS compared with mice subjected to IT-NaCl, with differences in general being most pronounced 72 h post instillation (Fig. 7a-d).

### Expression levels of mitochondrial fission and fusion genes in skeletal muscle in response to IT-LPS

**Fig. 8:**
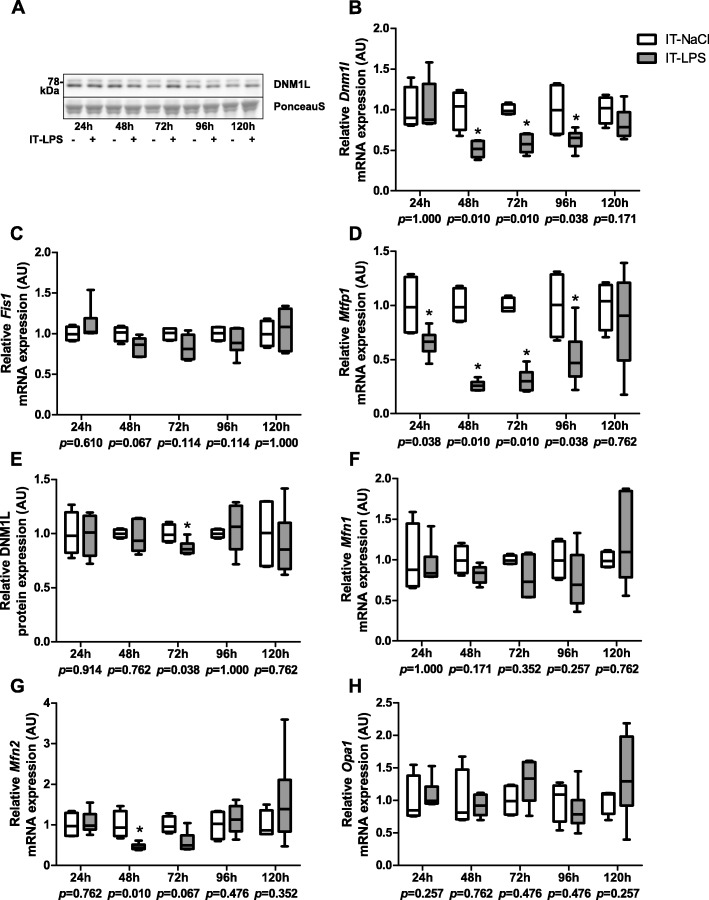
mRNA and protein expression of mitochondrial dynamics markers in m. gastrocnemius in response to IT-LPS. Fission related mRNA expression of *Dmn1l* (**b**), *Fis1* (**c**), *Mtfp1* (**d**), and protein expression of DMN1L (**e**) in m. gastrocnemius are depicted. Fusion related mRNA expression of *Mfn1* (**f**), and *Mfn2* (**g**), and *Opa1* (**h**) in m. gastrocnemius are depicted. A representative immunoblot and a representative part of Ponceau S staining are shown, with adjusted contrast equally applied to whole photograph (**a**). Samples were equally divided over multiple gels which were derived and processed in parallel. Data is presented as box plots indicating median and interquartile range, with whiskers indicating min and max. *p*-value and significance is depicted between groups within each time-point *p < 0.05

As mitochondrial fusion and fission are key events involved in mitochondrial remodelling by mitophagy and mitochondrial biogenesis, we next investigated mRNA transcript- and protein levels of proteins known to be involved in mitochondrial fusion and fission. As illustrated in Fig. 8, transcript abundance of the mitochondrial fission mediators dynamin-1-like (DNM1L) (48 h–96 h) and mitochondrial fission process 1 (MTFP1) (24 h–96 h) were significantly lower post instillation in mice subjected to IT-LPS compared with mice subjected to IT-NaCl while mitochondrial fission gene 1 (*Fis1*) transcript levels were unaltered (Fig. 8b-d). In addition, in line with mRNA expression levels, protein levels of DNM1L were significantly lower 72 h post instillation in the IT-LPS mice (Fig. 8e). With regard to mediators of mitochondrial fusion, only *Mfn2* transcript levels were lower 48 h post instillation in the IT-LPS mice, while no differences were found for both *Mfn1* and mitochondrial dynamin like GTPase (*Opa1*) (Fig. 8f-h).

### Mitochondrial content and AMPK signalling in skeletal muscle in response to IT-LPS

A secondary objective of this study was to explore whether the abovementioned alterations in abundance of molecular signalling constituents of mitophagy and mitochondrial biogenesis in muscle in response to an acutely-administered single bolus of IT-LPS were sufficient to induce changes in skeletal muscle mitochondrial content within the timeframe of the study. Therefore, we measured mtDNA copy number, protein levels of subunits of 4 mitochondrial OXPHOS complexes as well as activity levels of CS and HADH, 2 key mitochondrial enzymes respectively involved in the Krebs cycle and fatty acid β-oxidation. Moreover, as 5′ adenosine monophosphate-activated protein kinase (AMPK) is an important energy sensor and is known to mediate some of its functions through phosphorylationof acetyl-CoA carboxylase 1 (ACC1) (Ser79), we measured the phosphorylation status of both these proteins as markers of cellular energy status.

**Fig. 9:**
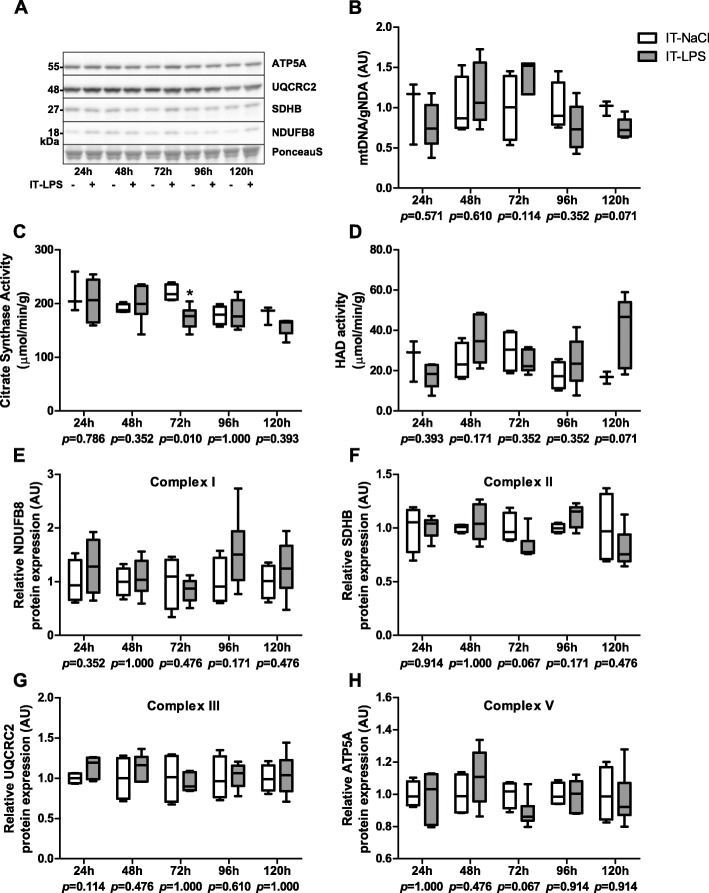
Expression and activity of key mitochondrial constituents in m. gastrocnemius in response to IT-LPS. mtDNA corrected for gDNA in m. gastrocnemius is depicted (**b**). CS (**c**) and HADH (**d**) activity in m. gastrocnemius are depicted. Protein expression of the following OXPHOS subunits in m. gastrocnemius is depicted; NDUFB8 of complex I (**e**), SDHB of complex II (**f**), UQCRC2 of complex III (**g**), and ATP5A of complex V (**h**). Representative immunoblots and a representative part of Ponceau S staining are shown, with adjusted contrast equally applied to whole photograph (**a**). Samples were equally divided over multiple gels which were derived and processed in parallel. Data is presented as box plots indicating median and interquartile range, with whiskers indicating min and max. *p*-value and significance is depicted between groups within each time-point *p < 0.05

**Fig. 10:**
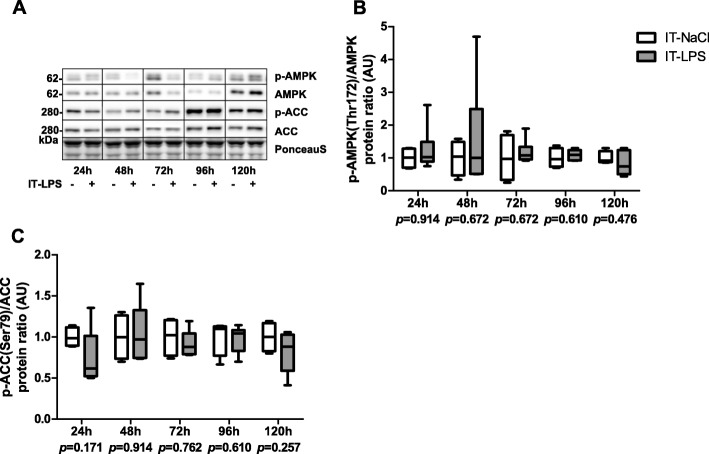
Expression of AMPK-signalling in m. gastrocnemius in response to IT-LPS. Ratios between phosphorylated protein and total protein of AMPK (**b**), and ACC (**c**) in m. gastrocnemius are depicted. Representative immunoblots and a representative part of Ponceau S staining are shown, with adjusted contrast equally applied to whole photograph (**a**). Samples were grouped by time-point over different gels. Data is presented as box plots indicating median and interquartile range, with whiskers indicating min and max. *p*-value and significance is depicted between groups within each time-point *p < 0.05

mtDNA copy number tended to be lower 120 h post instillation in mice subjected to IT-LPS compared with IT-NaCl (Fig. 9b). CS enzyme activity was lower 72 h post instillation, while no significant differences for HADH enzyme activity were found in mice subjected to IT-LPS compared with controls (Fig. 9c-d). Protein levels of subunits from OXPHOS complex II and V tended to be lower in the LPS group 72 h post instillation (Fig. 9f-h). No significant differences were found for p-AMPK(Thr172)/AMPK and p-ACC(Ser79)/ACC ratio (Fig. 10b-c).

### Comparison of molecular response of gastrocnemius and soleus muscle to 48 h IT-LPS

**Fig. 11:**
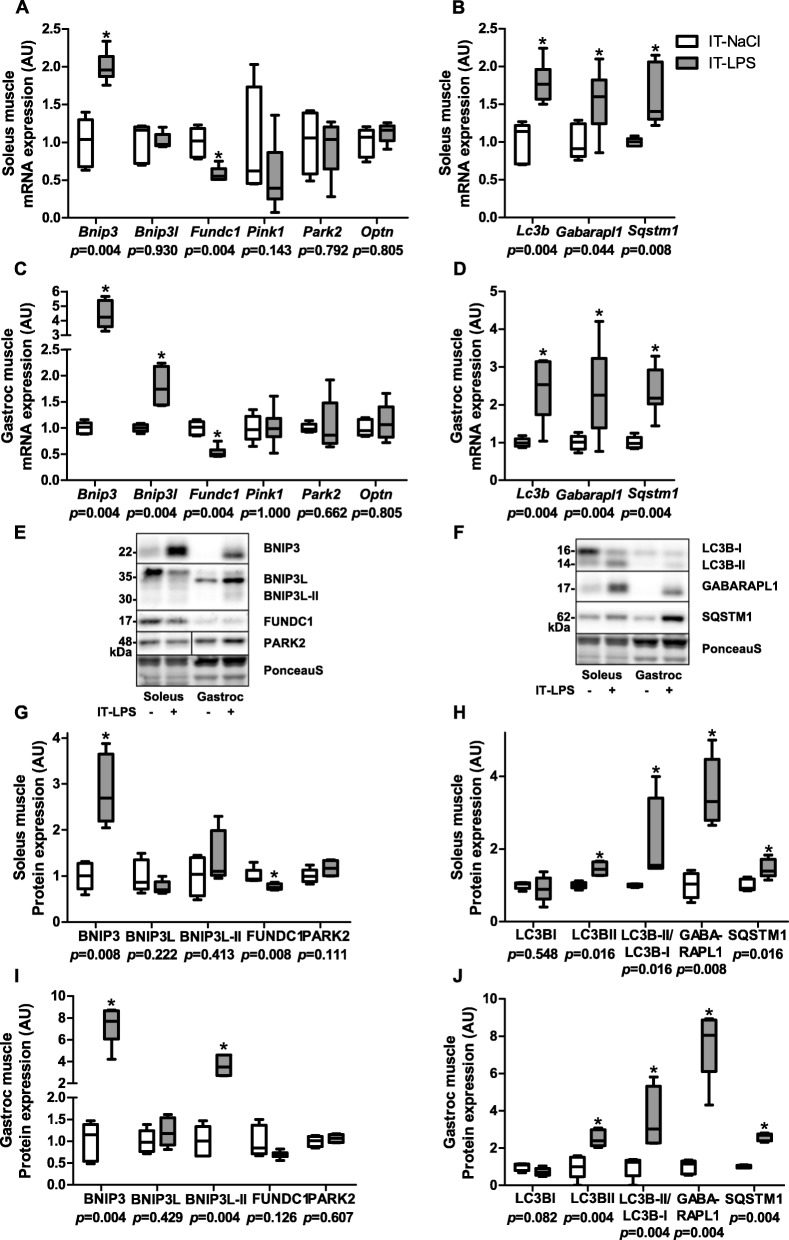
Mitophagy- and autophagy-signalling in m. soleus and gastrocnemius in response to IT-LPS. Mitophagy-, and autophagy-related mRNA expression levels in soleus and gastrocnemius muscle (**a**-**d**). Mitophagy-, and autophagy-related protein levels in soleus and gastrocnemius muscle (**g**-**j**). Representative immunoblots and a representative part of Ponceau S staining are shown, with adjusted contrast equally applied to whole photograph (**e**, **f**). Samples were run on one gel. Data is presented as box plots indicating median and interquartile range, with whiskers indicating min and max. *p*-value and significance is depicted between groups within each time-point *p < 0.05

**Fig. 12:**
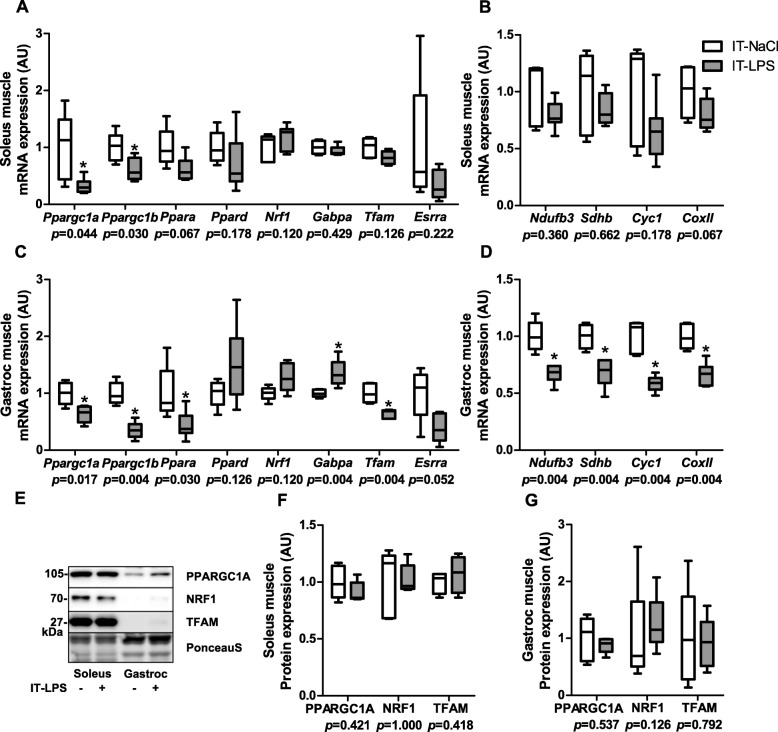
Mitochondrial biogenesis-signalling in m. soleus and gastrocnemius in response to IT-LPS. Mitochondrial biogenesis-, and oxphos-related mRNA expression levels in soleus and gastrocnemius muscle (**a**-**d**). Mitochondrial biogenesis-related protein levels in soleus and gastrocnemius muscle (**f**-**g**). Representative immunoblots and a representative part of Ponceau S staining are shown, with adjusted contrast equally applied to whole photograph (**e**). Samples were run on one gel. Data is presented as box plots indicating median and interquartile range, with whiskers indicating min and max. *p*-value and significance is depicted between groups within each time-point *p < 0.05

**Fig. 13:**
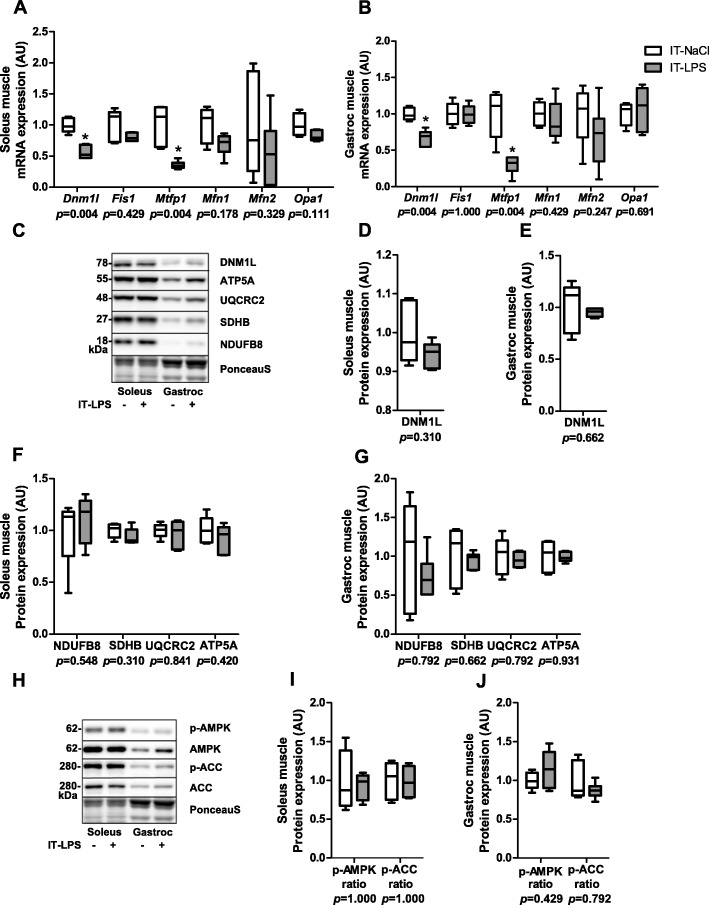
Mitochondrial dynamics, content, and AMPK-signalling in m. soleus and gastrocnemius in response to IT-LPS. Mitochondrial dynamics-related mRNA and protein expression levels in soleus and gastrocnemius muscle (**a-e**). Mitochondrial content-related protein levels in soleus and gastrocnemius muscle (**f-g**). AMPK-signalling-related protein ratios in soleus and gastrocnemius muscle (**i-j**). Representative immunoblots and a representative part of Ponceau S staining are shown, with adjusted contrast equally applied to whole photograph (**e**, **h**). Samples were run on one gel. Data is presented as box plots indicating median and interquartile range, with whiskers indicating min and max. *p*-value and significance is depicted between groups within each time-point *p < 0.05

To verify that the above described molecular response in the predominantly glycolytic gastrocnemius muscle is similar in the highly oxidative soleus muscle, we repeated all protein- and transcript quantification analyses in gastrocnemius and soleus muscle at 48 h post IT-LPS in an additional study. The IT-LPS instilled mice had lower body weight (19%, *p* = 0.004), soleus muscle weight (14%, *p* = 0.010) and gastrocnemius muscle weight (13%, *p* = 0.008). Although the amplitude of the responses differed between soleus and gastrocnemius muscle, most mitophagy-, autophagy- (Fig. 11), mitochondrial biogenesis- (Fig. 12), mitochondrial dynamics-, mitochondrial content-, and AMPK signalling- (Fig. 13) related markers showed similar patterns in both muscles in response to IT-LPS instillation.

Nevertheless, some differences in the molecular signalling patterns were detected between soleus and gastrocnemius muscle in response to IT-LPS instillation. Although BNIP3L transcript- and protein-expression were higher in the gastrocnemius in response to IT-LPS, no differences were detected in the soleus (Fig. 11a,c,g,i). Moreover, higher GABPA and lower TFAM, NUDUFB3, SDHB, CYC1 and COXII transcript levels were detected in gastrocnemius post IT-LPS, without corresponding changes in soleus muscle (Fig. 12a-d).

## Discussion

The current study shows that a single intra-tracheally instilled bolus of LPS results in a temporal modulation of mitophagy- and mitochondrial biogenesis-associated signalling in both oxidative and glycolytic skeletal muscle. These changes were associated with changes in upstream autophagy signalling, decreased transcript abundance of several OXPHOS complexes, and with reductions in transcript and protein levels of several proteins essentially involved in mitochondrial fission events. These changes were not associated with pronounced differences in skeletal muscle mitochondrial content. The currently reported changes in mitophagy- and mitochondrial biogenesis-associated signalling are associated with increased atrophy-signalling that was previously described in this study by our group [33].

Intra-tracheal instillation of LPS is a well-established model for bacterial-originating pulmonary inflammation-induced systemic inflammation. In detail, this model is characterized by both a prominent pulmonary inflammation, as identified by inflammatory cell influx and activation of inflammatory gene expression, and a systemic inflammation, evidenced by increased presence of tumor necrosis factor (TNF)-α, interleukin (IL)-1α, IL-1β, IL-6, chemokine (C-X-C motif) ligand (CXCL)1, RANTES and granulocyte-colony stimulating factor (G-CSF) in the circulation [27–30]. Moreover, the activation of inflammatory NF-κB signalling has been described in the skeletal muscle of this model [27]. These data are in line with the inflammatory profile reported in the current study, as we previously reported inflammatory cell recruitment, and increased gene-expression of pro-inflammatory cytokines and chemokines in these IT-LPS instilled lungs [33], and we now report increased levels of inflammatory gene expression in the gastrocnemius muscle of these mice.

It is evident that the expression of several mitophagy-related constituents is increased in muscle in response to IT-LPS in our study, peaking at 48 h after IT-LPS instillation. More specifically, both gene-expression of BNIP3 and BNIP3L, and protein levels of BNIP3 and BNIP3L-II (i.e. ±30 kDa band), are temporally higher after IT-LPS instillation. These changes are accompanied by higher gene-expression and protein levels of their down-stream autophagy-binding partners LC3B and GABARAPL1. In contrast, BNIP3L (i.e. ±35 kDa band) protein abundance was lower 72 h post-IT-LPS which may be indicative of mitophagy-mediated breakdown or post-transcriptional regulation. Although BNIP3 and BNIP3L have also been reported as mediators of cell death [35], the overlapping signalling pattern of autophagy markers, combined with the existing literature reporting mitophagy and increased BNIP3 and BNIP3L expression in skeletal muscle of experimental sepsis and pulmonary infection models [17–19, 22, 23], result in an expression pattern more indicative of mitophagy than apoptosis. In contrast to the transcriptionally-regulated BNIP3 and BNIP3L activation [36], FUNDC1 activation is mainly determined by post-translational modifications (e.g. phosphorylation) [37–40]. In line with this, we report decreased transcript levels of FUNDC1 at 72 h post IT-LPS, and decreased FUNDC1 protein abundance at 96 h post IT-LPS. However, as FUNDC1 can also rapidly be degraded by mitophagy-independent breakdown to protect the tissue from excess mitophagy during acute stress [41], interpretation of these data remains speculative. Although FUNDC1-mediated mitophagy has extensively been studied in several mammalian cell types during hypoxia [13, 39], its activation in skeletal muscle in response to inflammation has not been reported in vivo thus far.

Currently, we only report a marginal increase in PARK2 protein levels (96 h post IT-LPS) and no changes in PINK1 or PARK2 transcript levels, which suggests only a minor role for ubiquitin-mediated mitophagy in muscle of IT-LPS instilled mice. However, previous studies showed increased PARK2 protein- or gene-expression in skeletal muscle of experimental sepsis and pulmonary infection models [25, 42], suggesting that inflammation is able to modulate ubiquitin-mediated mitophagy in more severe inflammation models.

The alterations in mitophagy signalling coincided with prominent reductions in the transcript levels of PPARGC1B, PPARA and ESRRA, but were not accompanied by changes in PPARGC1A, NRF1 or TFAM transcript or protein expression. PPARGC1B, which promotes mitochondrial gene transcription when associated with NRFs and ESRRA or fatty acid oxidation when linked to PPARs [43], has been described to play an important role in determining the skeletal muscle oxidative phenotype [44]. In line with these results, we observed decreased transcript abundance of several nuclear- and mitochondrial-encoded OPXHOS subunits in response to IT-LPS.

In recent years, the processes of mitochondria breakdown, biogenesis and dynamics were found to be highly interconnected and interdependent. Indeed, mitophagy has been identified as a key process involved in mitochondrial biogenesis and remodelling in muscle [45–48], and the processes of mitophagy and mitochondrial biogenesis directly interact with regulators of mitochondrial dynamics [9, 37, 42, 49–52]. Although we did not measure actual fission or fusion events in the current study, our data indeed shows a modulation of the transcriptional regulation of mitochondrial dynamics, which is temporally associated with the reported changes in molecular signalling of mitophagy and mitochondrial biogenesis.

As we previously demonstrated a direct causal relationship between activation of NF-κB signalling and impairment of oxidative metabolism and the regulation thereof by the PPARGC1 network in cultured muscle cells [53, 54], it is feasible that the previously described IT-LPS-induced activation of muscle NF-κB signalling [27], is implicated in the impairments in the constituents controlling mitochondrial metabolism that we observed in the initial phase post IT-LPS. Also, autophagy-related gene-expression was found to be NF-κB-dependent in both IT-LPS and *i.p.* LPS inflammation models, while BNIP3 gene-expression was not [17, 27]. Although we reported increased muscular inflammatory signalling in response to IT-LPS instillation, NF-κB dependency was not examined in this study.

Mitochondrial impairments and decreased energy status could both be the result as well as the cause of increased mitochondrial breakdown. As we did not find profound changes in the skeletal muscle oxidative phenotype in our mice, in contrast to the literature from more severe experimental sepsis models [15, 17, 19, 20, 22], it is likely that the pulmonary inflammation-induced temporal shift in skeletal muscle mitochondrial homeostasis signalling is of insufficient amplitude to result in changes in mitochondrial content in the current study. This would also explain why, for example, OXPHOS subunit transcript abundance was temporally decreased post IT-LPS while no significant changes in OXPHOS protein levels were observed in response to IT-LPS. Moreover, as we report no changes in the phosphorylation of AMPK and ACC, it is unlikely that cellular energy status was severely compromised in our study. Combined, these data suggest that it is unlikely that decreased energy availability is the driver of the changes in molecular signalling of mitophagy and mitochondrial biogenesis in response to IT-LPS in our study, and that the changed signalling might be insufficient to result in robust changes in mitochondrial content.

Comparing mitochondrial homeostasis signalling patterns between relatively glycolytic gastrocnemius and highly oxidative soleus muscle showed that soleus muscle has a lacking increase in BNIP3L gene- and protein-expression, and a less pronounced decrease in transcriptional regulation of mitochondrial biogenesis. As the direction of the remaining expression patterns were comparable between muscle types, and the observed differences were on average more pronounced in gastrocnemius muscle, our results are in line with previous studies in pulmonary-inflammation and experimental sepsis models [17, 25], and suggest a conserved molecular response to pulmonary-inflammation induced systemic inflammation in different muscle types.

Although we report data on a comprehensive set of proteins involved in the execution and regulation of mitophagy, autophagy, mitochondrial biogenesis, and mitochondrial dynamics measured in an extensive time-course after instillation of IT-LPS, we are aware that our study has some limitations. First, we quantified abundance of many key players heavily involved in, and indicative of mitophagy initiation but no actual mitophagy or autophagy flux was measured in our samples. Therefore, the increased protein abundance of the mitophagy and autophagy-related proteins might also be the result of impaired breakdown instead of increased synthesis. Moreover, we describe associations between changes in the processes of autophagy/mitophagy and mitochondrial biogenesis, but the causality of these associations remains unclear in our study. In addition to the known decrease in physical activity, it has been shown that food intake is reduced in this model [33]. As decreased physical activity and food intake both have been shown to be able to modulate mitophagy and mitochondrial biogenesis in muscle [55, 56], a potential contribution of these aspects to the changes we observed in muscle of IT-LPS-instilled animals cannot be discarded. Moreover, a previous study showed that reduced food intake accounts for 60% of the loss in body weight and 55% of the loss of muscle weight 24 h after IT-LPS instillation [27]. Therefore, as we did not include a pair-fed group, the relative contributions of starvation, changes in physical activity, and inflammation to the activation of the autophagy/mitophagy pathways in peripheral muscle cannot be discerned in the current study.

Despite these limitations, this study provides the most comprehensive overview of changes in pathways controlling mitochondrial breakdown, biogenesis, and dynamics in peripheral muscle in vivo in response to IT-LPS instillation to date, which, in combination with the previous study [33], suggests a coordinated temporal regulation of these processes in response to IT-LPS.

## Conclusions

The current study reports a comprehensive overview of changes in key proteins controlling mitophagy, mitochondrial biogenesis and dynamics, without corresponding changes in mitochondrial content, in oxidative and glycolytic peripheral muscle in response to acute pulmonary inflammation induced by IT-LPS instillation. These results suggest that mitophagy is activated and mitochondrial biogenesis is decreased in skeletal muscle exposed to pulmonary-inflammation induced systemic inflammation. The timeframe of these alterations suggests that they could be part of a coordinated physiological response of skeletal musculature to an inflammatory insult. These results provide valuable insights in the regulation of mitochondrial homeostasis after acute pulmonary inflammation and may contribute to the development of future mitochondrial health-preserving therapies.

## Data Availability

The datasets used and/or analysed during the current study are available from the corresponding author on reasonable request.

## Abbreviations

ACC: Acetyl-CoA Carboxylase
AMPK: AMP-activated protein kinase
BNIP3: BCL2/adenovirus E1B 19 kDa protein interacting protein 3
BNIP3L: BCL2/adenovirus E1B 19 kDa protein interacting protein 3 like
COPD: Chronic Obstructive Pulmonary Disease
CS: Citrate synthase
CXCL: Chemokine (C-X-C motif) ligand
DNM1L: Dynamin-1-like
ESRRA: Estrogen related receptor, alpha
FIS1: Mitochondrial fission gene 1
FUNDC1: FUN14 domain containing 1
GABARAPL1: Gamma-aminobutyric acid (GABA) A receptor-associated protein-like 1
GABPA: GA repeat binding protein, alpha
G-CSF: Granulocyte-colony stimulating factor
gDNA: Genomic DNA
HADH: β-hydroxyacyl-CoA dehydrogenase
i.p.: Intraperitoneal
IL: Interleukin
IT-LPS: Intra-tracheally instilled lipopolysaccharide
LC3B: Microtubule-associated protein 1 light chain 3 beta (MAP 1LC3B)
MFN: Mitofusin
mtDNA: Mitochondrial DNA
MTFP1: Mitochondrial fission process 1
NF-κB: Nuclear factor kappa B
NRF1: Nuclear respiratory factor 1
OPA1: Mitochondrial dynamin like GTPase
OPTN: Optineurin
OXPHOS: Oxidative phosphorylation
PARK2: Parkin
PINK1: PTEN induced putative kinase 1
PPAR: Peroxisome proliferative activated receptor
PPARGC1: PPAR gamma, coactivator 1
SQSTM1: Sequestosome-1
TFAM: Transcription factor A, mitochondrial
TNF: Tumor necrosis factor
